# PET/CT Radiomics Integrated with Clinical Indexes as a Tool to Predict Ki67 in Breast Cancer: a Pilot Study

**DOI:** 10.1007/s13139-024-00896-9

**Published:** 2024-11-29

**Authors:** Dawei Li, Hui Ding, Yuting Liao, Xiao Yu, Youmin Guo, Cong Shen

**Affiliations:** 1 Department of Positron Emission Tomography/Computer Tomography, The First Affiliated Hospital of Xi’an Jiaotong University, 277 Yanta West Road, Xi’an, Shaanxi 710061 China; 2 Department of Radiology, The First People’s Hospital of Jinzhou District, Dalian, Liaoning P.R. China; 3 Clinical & Technical Support, Philips Healthcare, Shanghai, China

**Keywords:** PET/CT, Radiomics, Breast cancer, Ki67, Predictive model

## Abstract

**Objective:**

This study aims to assess the value of radiomics features integrated with clinical characteristics for estimating Ki67 expression in patients with breast cancer (BC).

**Methods:**

In total, 114 patients with BC performed ^18^F-FDG PET/CT scans. Patients were randomly assigned to a training set (*n* = 79, 55 cases of Ki67 + and 24 cases of Ki67-) and a validation set (*n* = 35, 24 cases of Ki67 + and 11 cases of Ki67-). Thirteen clinical characteristics and 704 radiomics features were extracted, and 4 clinical and 8 radiomics features were selected. Three models were developed, including the clinical model, the radiomics model, and the combined model. Model performance was evaluated using the ROC curve, and clinical utility was assessed through decision curve analysis (DCA).

**Results:**

The N stage, tumor morphology, SUVmax, and the longest diameter significantly differed between Ki67 + and Ki67- groups (all *P* < 0.05). Eight radiomics features were selected for the radiomics model. The area under the curve of the combined model in the training and test group was 0.90 (95% CI: 0.82∼0.97) and 0.81 (95% CI: 0.64∼0.99), respectively. The combined model significantly outperformed both the radiomics model and the clinical model alone (*P* < 0.05). The DCA curve demonstrated the superior clinical utility of the combined model compared to the clinical model and radiomics model.

**Conclusions:**

PET/CT image-based radiomics features combined with clinical features have the potential to predict Ki67 expression in BC.

**Supplementary Information:**

The online version contains supplementary material available at 10.1007/s13139-024-00896-9.

## Introduction

Breast cancer (BC) is a heterogeneous malignancy for women, and BC represents the second cause of cancer-related death among women [1]. Ki67 is the most commonly used biomarker for evaluating the proliferative index of BC. Several studies have found that high Ki67 expression is associated with an elevated relapse rate and worse survival in BC [2–5]. Other studies have demonstrated the clinical validity of Ki67 as a predictive marker in neoadjuvant settings [6]. Nevertheless, tumor biology characterization is an invasive procedure; thus, a non-invasive technique for Ki67 evaluation would be worthwhile.

^18^F-fluorodeoxyglucose (^18^F-FDG) positron emission tomography/computed tomography (PET/CT) is widely and routinely used in BC staging [7]. ^18^F-FDG PET/CT can provide extra metabolic information compared with traditional imaging modalities. Standard semi-quantitative imaging variables obtained from ^18^F-FDG PET/CT, such as the maximum standardized uptake value (SUVmax), the mean standardized uptake value (SUVmean), and the metabolic tumor volume (MTV), have been shown to correlate with tumor aggressiveness and patient outcomes in BC [8–11]. However, those quantitative variables could not fully capture BC heterogeneity.

Radiomics, an approach that aims to extract quantitative features from medical imaging to quantify lesion heterogeneity and texture, holds promise in addressing clinical challenges in monitoring disease progression [12]. Recent reports indicate that features obtained from ^18^F-FDG PET/CT are associated with the molecular subtypes [13–15], but there is limited evidence of their roles with Ki67.

The main objective of this study was to investigate the value of radiomics features derived from ^18^F-FDG PET/CT combined with clinical characteristics in predicting Ki67 in patients with BC.

## Materials and Methods

### Study Population

This retrospective study was conducted at the First Affiliated Hospital of Xi ‘an Jiaotong University (NCT05826197), and the study protocol was approved by the Ethics Committee of Xi ‘an Jiaotong University (IRB-SOP-AF-16).

Between November 2016 to June 2020, a total of 129 female patients with BC who underwent ^18^F-FDG PET/CT examinations were retrospectively studied. Inclusion criteria were: (1) BC confirmed through preoperative puncture or postoperative pathology; (2) underwent ^18^F-FDG PET/CT examination; and (3) available Ki67 expression. Exclusion criteria were: (1) the primary lesion was too small to detect by ^18^F-FDG PET/CT or occult BC patients (*n* = 5); (2) diffuse lesion on unilateral side or multifocal lesions in bilateral side (*n* = 4); (3) benign breast lesions (*n* = 1); and (4) neo-adjuvant chemotherapy or anti-tumor treatment performed before imaging (*n* = 4).

**Fig. 1:**
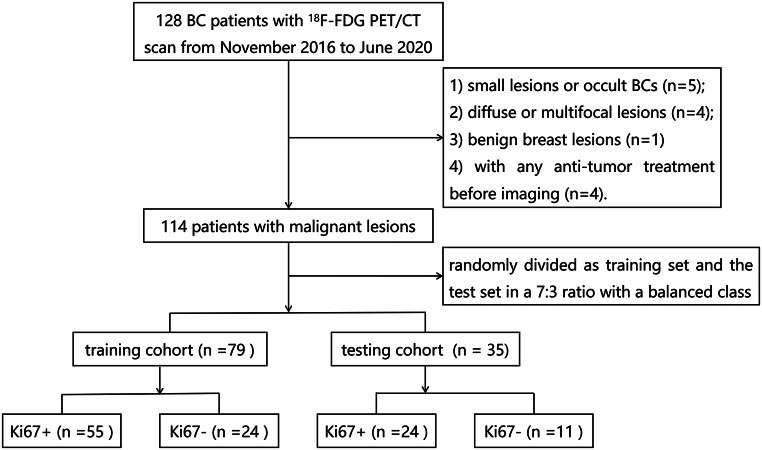
The workflow of the BC patient selection. BC, breast cancer; FDG, fluorodeoxyglucose

A flowchart of this process is shown in Fig. 1. A total of 114 patients were enrolled in the study. Patients were randomly assigned to a training group (79 patients) and a test group (35 patients) at a ratio of 7 to 3 using a stratified sampling method. The age, location (right side or left side) of the BC, and the menopausal status (premenopausal or during and beyond menopausal) of the patient were recorded.

### Immunohistochemistry

Formalin-fixed paraffin-embedded tissue samples from BC cases were used for Ki67 assessment by an experienced pathologist blinded to the PET/CT results. Ki67 levels were divided into the 0.5%, 10%, 15%, 20%, 30%, 40%, 50%, 60%, 70%, 80%, 90%, and 100% groups according to the degree of expression, and an expression index ≥ 20% was considered positive.

### PET/CT Data Acquisition

All examinations were performed using a 64-detector scanner (Gemini TF PET/CT, Philips, Netherlands). ^18^F-FDG was synthesized using a small cyclotron (GE MINItrace) and an FDG synthesis module. Radiochemical purity was > 99%. Both endotoxin and bacteriological tests were negative, which met the radio-pharmaceutical requirements.

The patients fasted for over six hours before intravenous injection of ^18^F-FDG (3.7 MBq/kg). The fasting blood glucose level should be lower than 12.0 mmol/L. After resting for 60 min, the patients underwent whole-body PET/CT. The scan scope was from the top of the skull or the level of the first thoracic vertebra to the upper femur. PET collects 6–10 beds with 1.5 min/bed. CT scans (tube voltage, 120 kV; automatic milliampere second; matrix, 512 × 512; layer thickness, 5 mm) were performed for lesions’ location and attenuation correction of the PET image. MIP (Maximum Intensity Projection), PET, CT, and fusion images were displayed on the Extended Brilliance Workstation (EBW) workstation.

### Image Analysis

Manually defined features, including tumor morphology (regular or irregular), necrosis (with or without), and calcification (with or without), as well as the N (N0, or N1) and M (M0, or M1) stages, were determined in a double-masked manner by two experts (Dawei Li, reader 1; Cong Shen, reader 2) with more than ten years of PET/CT interpretation experience. Any disagreement between the two radiologists was resolved by another experienced radiologist.

**Fig. 2:**
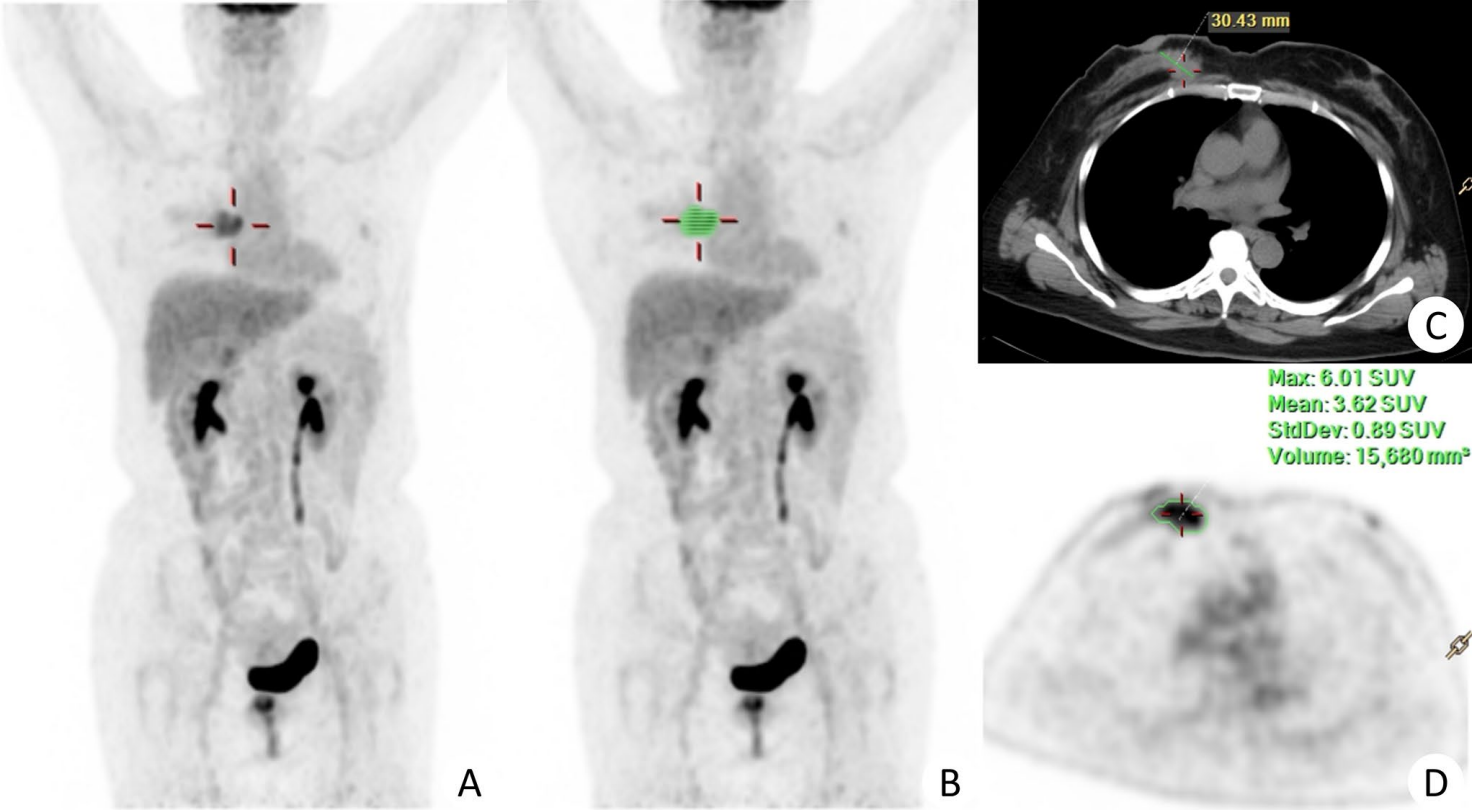
The delineation of the VOI of BC. A female, 56 years old, with breast cancer on the right side, underwent an ^18^F-FDG PET/CT scan. (**A**) (MIP) showed a high uptake of ^18^F-FDG of the cancer. The VOI was segmented with a 40% SUVmax on the EBW workstation (**B**). The longest diameter of the cancer was measured on the maximal horizontal position and was 30.43 mm (**C**). The SUVmax, SUVmean, SD, and MTV were 6.01, 3.62, 0.89, and 15.680mm^3^ (**D**)

The longest diameter (mm) was measured at the maximal horizontal position. The volume of interest (VOI) was automatically delineated with a 40% maximum standardized uptake value (SUVmax) as the threshold on the EBW workstation. The VOIs were reviewed, and manual correction was allowed when the tumor border was unsatisfied. SUVmax, mean SUV (SUVmean), standard deviation (SD) of SUV, and metabolic tumor volume (MTV) were calculated, see Fig. 2.

### Feature Extraction

The VOIs were saved as *nifty* files. Radiomics feature extraction was implemented using the Philips Radiomics Tool (Philips Healthcare, China), and the core feature calculation was based on pyRadiomics (3.0.1) [16]. A total of 704 three-dimensional (3D) radiomics features were extracted, including the original features (*n* = 83), wavelet transform (*n* = 552), and logarithm transform (*n* = 69). The original features consist of shape-based features (*n* = 14), first-order statistics features (*n* = 18), gray-level run length matrix (GLRLM) (*n* = 16), neighboring gray-tone difference matrix (NGTDM) (*n* = 5), gray-level dependence matrix (GLDM) (*n* = 14), and gray-level size zone matrix (GLSZM) (*n* = 16). The wavelet transform (8 decompositions per level) and logarithm transform were applied to each feature in the category of the first-order statistics features, GLRLM, NGTDM, GLDM, and GLSZM. The categories and the names of all the 704 radiomics features were summarized in Supplementary Table 1. Details of the feature extraction definition are shown on the website (https://pyradiomics.readthedocs.io/en/latest/features.html#). There were no missing data for clinical and radiomics features.

### Feature Reduction

In the training group, for all thirteen clinical features (general characteristics and manually measured features), a univariate logistic regression analysis test was applied to select features with a *P* value < 0.1 for the subsequent analysis. The maximum relevance minimum redundancy (mRMR, top 30 features were selected) and least absolute shrinkage and selection operator (LASSO) with the optimal **λ** were applied to choose the most discriminative radiomics features for predicting Ki67 status. Mean squared error (MSE) was used in the feature reduction. After that, the Spearman test with a threshold of 0.8 was used for the clinical features and radiomics features, respectively, to delete the collinear features. The one with the highest correlation was retained at last. The clinical model was built using the selected clinical features. The radiomics model was built using selected radiomics features and expressed as Radscore. Then, the selected clinical features and radiomics features were combined, and the LASSO regression model was applied again to delete the collinear features.

### Statistical Analysis, Model Construction, and Evaluation

Data were analyzed using SPSS^®^ v. 25.0. (IBM Corp., New York, NY, USA) and python V 3.9 (URL https://www.python.org/). Continuous variables with abnormal distributions were expressed as median [25%, 75%] and were tested using the Mann–Whitney U test. Categorical variables were compared using the χ^2^ test or Fisher’s exact test. Statistical significance was set at *p* < 0.05.

Three models, including the clinical, radiomics, and combined clinical-radiomics, were constructed using a logistic regression model. The predicted probability of clinical, radiomics, and combined clinical-radiomics models was defined as clinical score, Radscore, and combined score, respectively. The formula is expressed as a linear regression model by multiplying and summing the feature values and related weight coefficients and then passing the result through a sigmoid transformation. Models were assessed using the receiver operating characteristic (ROC) curve to discriminate Ki67 + cases from Ki67- cases. Indexes were calculated, including area under the curve (AUC), sensitivity, specificity, and accuracy (ACC). The DeLong test was used to compare the differences between ROC curves.

The calibration curves and Hosmer-Lemeshow test were utilized to assess the agreement between predicted and actual probabilities of various models, and *a P value > 0.05 means* good consistency. A nomogram was constructed to visualize the predictive model for Ki67 expression. The decision curve analysis (DCA) was used to determine the nomogram’s clinical usefulness by quantifying the net benefits at different threshold probabilities.

## Results

### Clinical Characteristics

The clinical characteristics of the patients are summarized in Table 1. The two cohorts had no significant differences regarding age, menstruation status, lesion location, or M stage (*P* = 0.521, *P* = 0.567, *P* = 0.482 and *P* = 0.102, respectively). Patients in the group with Ki67 positive showed irregular tumor morphology, higher N stage, higher longest diameter, higher SUVmax, higher SUVmean, higher SD, and higher MTV than those of patients in the group with Ki67 negative (*P* = 0.003, *P* < 0.001, *P* = 0.012, *P* < 0.001, *P* < 0.001, *P* < 0.001, and *P =* 0.031, respectively) (Table 1).

**Table 1:** Clinical characteristics and manual quantitative analysis of primary tumors on PET/CT images

Parameter	All (*n* = 114)	Ki67- (*n* = 35)	Ki67+ (*n* = 79)	Z/χ^2^	*p*
Age (year)	49.0 [40.0; 56.0]	51.0 [40.5;60.5]	49.0 [39.5;54.0]	0.549^a^	0.521
Menstrual status				0.420^b^	0.517
Premenopausal	67 (58.8%)	19 (54.3%)	48 (60.7%)		
During and beyond menopausal	47 (41.2%)	16 (45.7%)	31 (39.2%)		
Lesion location				0.495^b^	0.482
Left	61 (53.5%)	17 (48.6%)	44 (55.7%)		
Right	53 (46.5%)	18 (51.4%)	35 (44.3%)		
Tumor morphology				9.048^b^	**0.003**
Quasi circular	64 (56.1%)	27 (77.1%)	37 (46.8%)		
Irregular	50 (43.9%)	8 (22.9%)	42 (53.2%)		
Necrosis				--	0.310
No	110 (96.5%)	35 (100%)	75 (94.9%)		
Yes	4(3.51%)	0 (0.00%)	4 (5.06%)		
Calcification				0.213^b^	0.645
No	109 (95.6%)	33 (94.3%)	76 (96.2%)		
Yes	5 (4.39%)	2 (5.71%)	3 (3.80%)		
N stage				15.584^b^	**< 0.001**
N0	47 (41.2%)	24 (68.6%)	23 (29.1%)		
N1	67 (58.8%)	11 (31.4%)	56 (70.9%)		
M stage				--	0.102
M0	102 (89.5%)	34 (97.1%)	68 (86.1%)		
M1	12 (10.5%)	1 (2.86%)	11 (13.9%)		
The longest diameter (mm)	18.0 [13.1;29.0]	15.1 [11.9;18.4]	20.5 [14.9;35.5]	2.520^a^	**0.012**
SUV_max_	6.32 [4.71;9.36]	5.32 [3.60;6.47]	7.29 [5.28;11.0]	5.400^a^	**< 0.001**
SUV_mean_	3.67 [2.78;5.33]	2.95 [1.93;3.85]	4.29 [2.92;5.87]	5.400^a^	**< 0.001**
SD	1.03 [0.55;1.59]	0.57 [0.40;1.15]	1.15 [0.69;1.83]	5.400^a^	**< 0.001**
MTV (mm^3^)	5824 [2896;13216]	3072 [2112;8192]	6912 [3424;16000]	2.160^a^	**0.031**

*Note* a, Z test; b, χ^2^; --, Fisher exact probability test; SUV, standardized uptake value; SD, standard deviation, MTV, metabolic tumor volume

### Results of Feature Extraction

Table 2 shows the selected clinical features after applying univariate analysis, the Spearman test, and the features’ weight in the logistic regression model. Thus, the clinical score = 1.203 * N stage + 0.350 * Tumor morphology + 0.244 * SUVmax + 0.037 * The longest diameter − 2.257.

**Table 2:** Retained clinical features and its weight in the clinical logistic regression model

Method	Univariate logistic regression	Spearman test	Weight
Clinical features	N stage	N stage	1.203
	SUVmax	SUVmax	0.244
	SUVmean	The longest diameter	0.037
	The longest diameter	Tumor morphology	0.350
	Tumor morphology		
Intercept			-2.257

SUVmax: maximum standardized uptake value; SUVmean: mean standardized uptake value

$$\begin{aligned}&\text{The\:predicted\:probability\:of\:the\:clinical\:model}\cr&\quad=\frac{1}{1+{e}^{-clinical\:score}}\end{aligned}$$

**Fig. 3:**
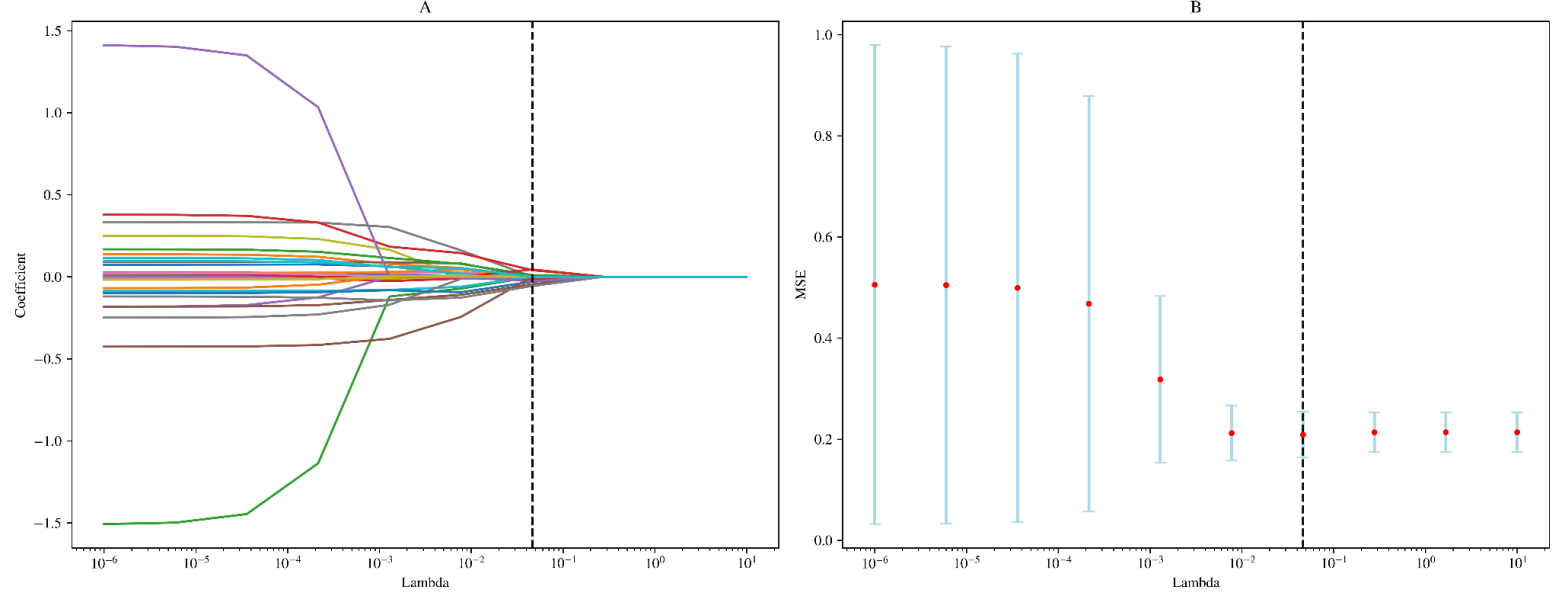
Radiomics feature selection using LASSO in the training group. Each curve represents the change trajectory of one variable coefficient in **A**. **B** shows the cross-validation curve of LASSO regression. The optimal penalization coefficient lambda (λ) was chosen using 10-fold cross-validation. Dotted vertical lines were drawn using the minimum criteria and the one standard error of the minimum criteria. Mean squared error (MSE) was used to evaluate the model’s effectiveness in the training group

For 704 radiomics features, mRMR was used firstly to select the top 30 features; then, LASSO was applied for further feature reduction, see Fig. 3. Table 3 shows the eight selected radiomics features after using mRMR and LASSO, the Spearman test, and the features’ weight in the logistic regression model. Thus, the Radscore = (-0.215) * HHH_glszm_GrayLevelNonUniformityNormalized + (-0.472) * HHL_ngtdm_Contrast + 0.313 * LHH_glszm_SmallAreaLowGrayLevelEmphasis + (-0.418) * HLL_glszm_SmallAreaEmphasis + 0.319 * LHH_gldm_DependenceVariance + 0.432 * LLL_firstorder_90Percentile + (-0.286) * log-sigma-6-0-mm-3D_glrlm_ShortRunHighGrayLevelEmphasis + (-0.517) * LHL_glrlm_RunEntropy + 1.036.

**Table 3:** Retained radiomics features and its weight in the radiomics logistic regression model

Method	mRMR and LASSO	Spearman test	Weight
Radiomics features	LHL_glrlm_RunEntropy	LHL_glrlm_RunEntropy	-0.517
	HHL_ngtdm_Contrast	HHL_ngtdm_Contrast	-0.472
	HLL_glszm_SmallAreaEmphasis	HLL_glszm_SmallAreaEmphasis	-0.418
	HHH_glszm_GrayLevelNonUniformityNormalized	HHH_glszm_GrayLevelNonUniformityNormalized	-0.215
	LHH_glszm_SmallAreaLowGrayLevelEmphasis	LHH_glszm_SmallAreaLowGrayLevelEmphasis	0.313
	LHH_gldm_DependenceVariance	LHH_gldm_DependenceVariance	0.319
	LLL_firstorder_90Percentile	LLL_firstorder_90Percentile	0.432
	log-sigma-6-0-mm-3D_glrlm_ShortRunHighGrayLevelEmphasis	log-sigma-6-0-mm-3D_glrlm_ShortRunHighGrayLevelEmphasis	-0.286
	original_firstorder_90Percentile		
Intercept			1.036

mRMR: maximum relevance minimum redundancy; LASSO: least absolute shrinkage and selection operator

**Fig. 4:**
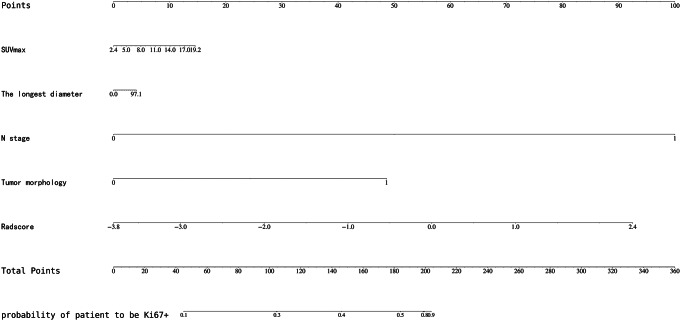
The nomogram was developed by combining radiomic and clinical features. Four selected clinical features were enrolled in the nomogram individually, while the eight selected radiomics features were enrolled as a comprehensive index, Radscore. The predicted value for testing was the probability of the patient being Ki67 positive. *Abbreviations* SUVmax, maximum standardized uptake value

$$\begin{aligned}&\text{The\:predicted\:probability\:of\:the\:Radscore\:model}\cr&\quad=\frac{1}{1+{e}^{-Radscore}}\end{aligned}$$

The mean values of eight radiomics features were compared between two groups and shown in Supplementary Table 2.

### Construction of a Combined Model for Predicting Ki67

The selected clinical features and Radscore were combined to construct a logistic regression model, and the results were visualized in the nomogram in Fig. 4. The combined score = 1.826 * N stage + 0.621 * Tumor morphology + 0.105 * SUVmax + 0.018 * The longest diameter + 1.329 * Radscore − 2.417.

$$\begin{aligned}&\text{The\:predicted\:probability\:of\:combined\:model}\cr&\quad=\frac{1}{1+{e}^{-combined\:score}}\end{aligned}$$

**Fig. 5:**
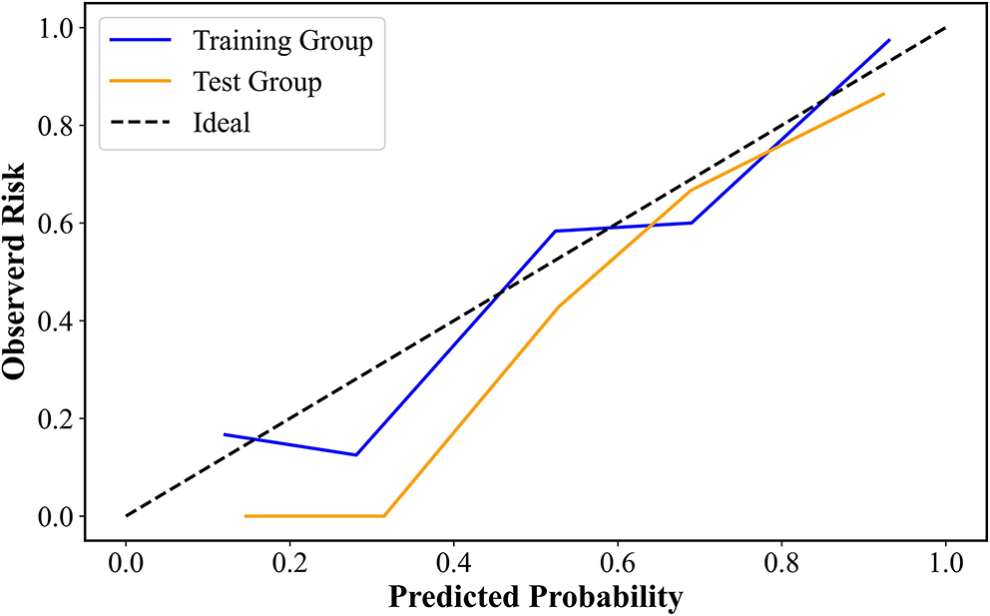
Calibration curves with the Hosmer–Lemeshow test of the nomogram. The calibration curves show the consistency between the predicted ki67 status and the real observed ki67 status for the combined model in the training and test groups. The x-axis represents the expected risk of ki67 status, and the y-axis represents the actual ki67 status

The calibration curve of the combined model for Ki67 expression prediction probability shows good accordance between prediction and observation in the training and test cohort (*P* > 0.05), shown in Fig. 5.

### Evaluation of the Performance of the Three Models

**Fig. 6:**
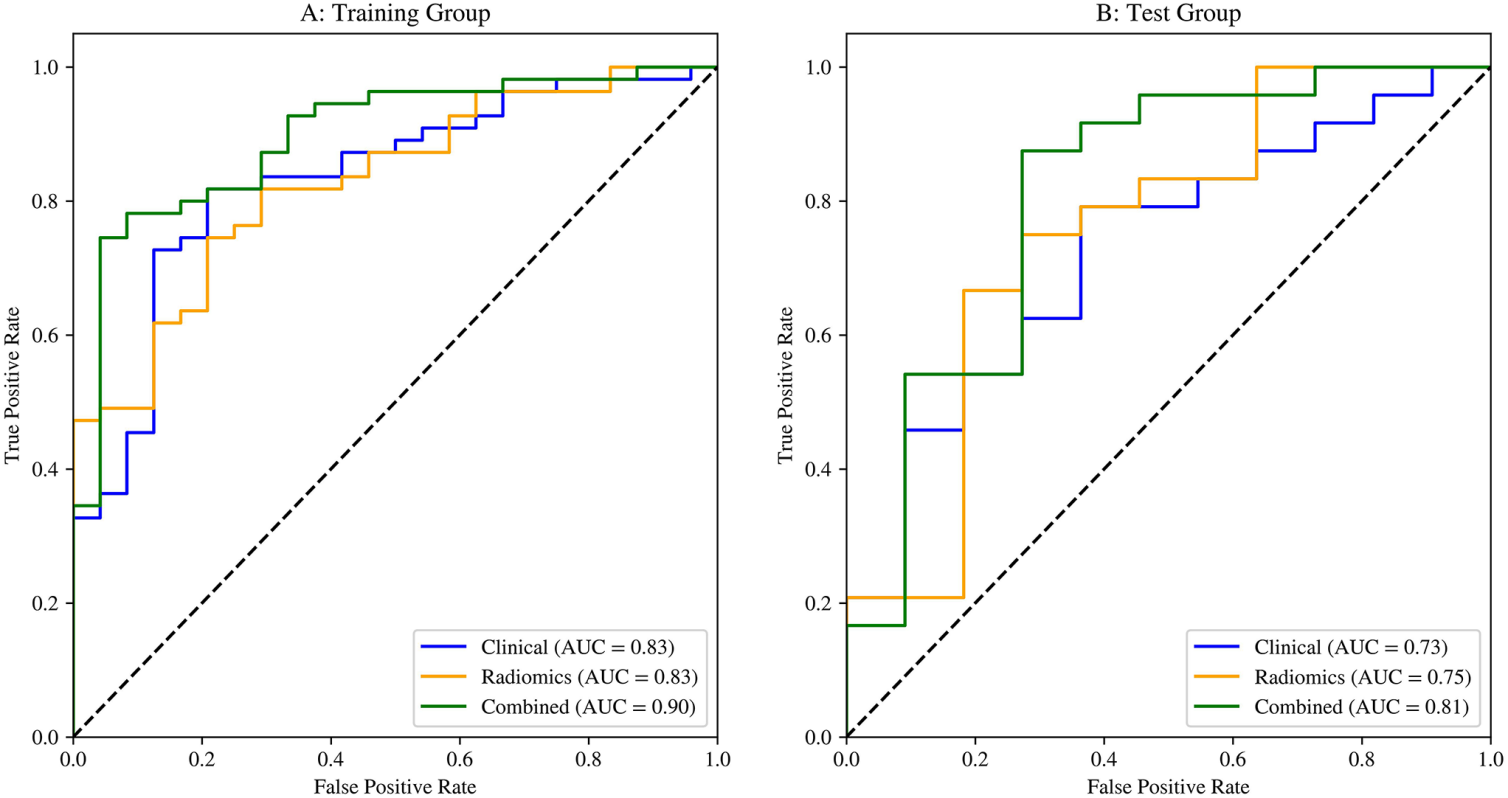
ROC of the three models in the training cohort and testing cohort

The combined model in both training and testing groups showed satisfactory performance in predicting Ki67 (Table 4). The AUC values for the combined model of the training and test groups were 0.90 (95% CI: 0.82–0.97) and 0.81 (95% CI: 0.64–0.99), respectively, see Fig. 6.

**Table 4:** The performance of three models in the training set and test set

Models	Data set	AUC (95%CI)	ACC	Sensitivity	Specificity
Clinical model	Training set	0.83[0.74 0.93]	0.81	0.82	0.79
Clinical model	Test set	0.73[0.55 0.91]	0.74	0.79	0.64
Radiomics model	Training set	0.83[0.74 0.92]	0.76	0.75	0.79
Radiomics model	Test set	0.75[0.56 0.95]	0.71	0.88	0.36
Combined model	Training set	0.90[0.82 0.97]	0.81	0.75	0.96
Combined model	Test set	0.81[0.64 0.99]	0.80	0.83	0.73

AUC: area under the curve; CI: confidence interval; ACC: accuracy. The combined model represents the model that combined the clinical and radiomics features

The AUC values of the clinical, radiomics, and radiomics models were compared using the DeLong test. It was found that the combined model significantly outperformed the radiomics model (*P* = 0.049) alone in the test group. The combined model also outperformed the clinical model alone, although without significance ((*P* = 0.059).

**Fig. 7:**
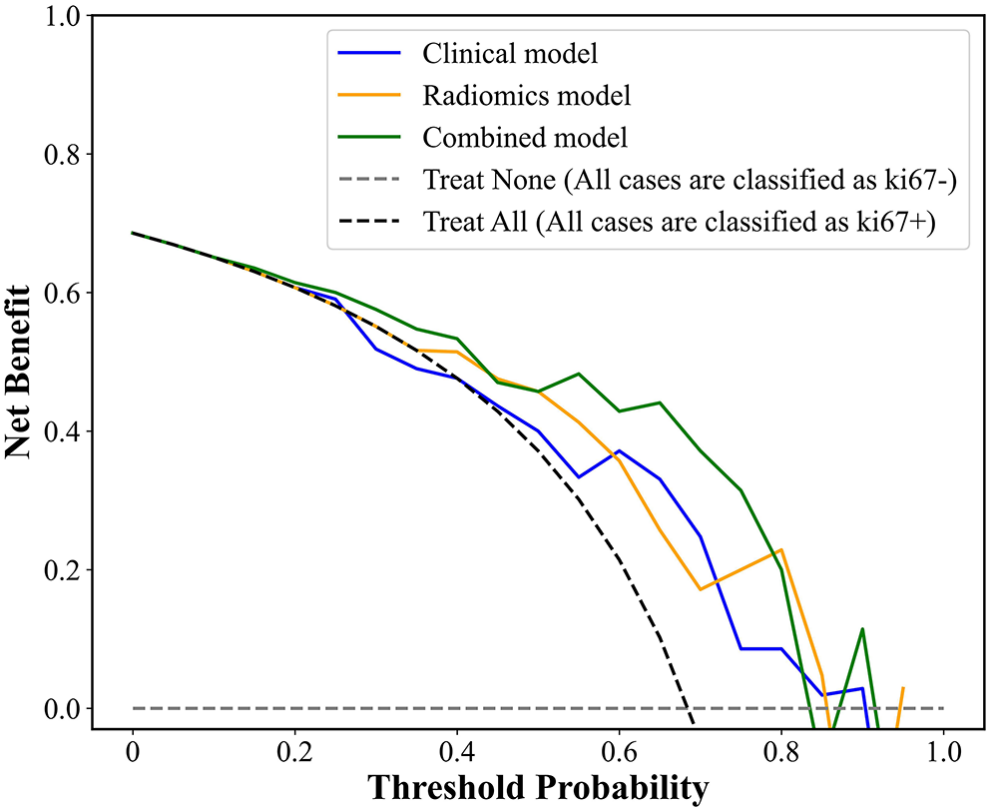
Decision curve analysis (DCA) of the test group. The y-axis measures the net benefit. The dotted line represents the assumption that all cases were classified as Ki67+ (Treat-all) or Ki67- (Treat-none). The nomogram added more benefits than either the treat-all or the treat-none scheme

The DCA curve was used to assess the clinical usefulness of the nomogram. Under the most threshold probability, using the combined model to predict Ki67 status added more benefit compared to both the treat-all and treat-none schemes, within a threshold probability range of 0.22 to 1, see Fig. 7. The DCA curve showed the advantages of the integrated model over the clinical and radiomics models.

## Discussion

Breast cancer is a heterogeneous malignancy with high mortality and morbidity. Ki67 is essential for identifying patients with worse survival and for neoadjuvant treatment. Radiomics is a newly introduced image-analysis method involving high-throughput feature extraction from radiographic images, which is promising for predicting tumor heterogeneity because of its noninvasive and repeatable properties.

Our study showed that the group with Ki-67 positive had a significantly higher longest diameter, SUVmax, SUVmean, SD, and MTV compared with the group with Ki-67 negative on ^18^F-FDG PET/CT images (*P* = 0.012, *P* < 0.001, *P* < 0.001, *P* < 0.001, and *P =* 0.031, respectively), as the previous studies [17–19]. Because the SUV-based parameters SUVmax, SUVmean, SD, and MTV) are expected to be highly correlated and give similar information; thus, only one of the SUV parameters has been used in the clinical model. Eight radiomics features were selected. Therefore, we constructed a nomogram incorporating eight radiomics and four clinical features for predicting Ki67 expression in BC. The validation of the nomogram showed that it had good discriminative and calibration capabilities. The AUC of the training and test groups were 0.90 (95% CI: 0.82–0.97) and 0.81 (95% CI: 0.64–0.99), respectively. The DeLong test found that the combined model significantly outperformed the radiomics and clinical models alone. Also, the DCA displayed a more significant net benefit for the combination of clinical and radiomics features than the one alone. This strategy may have clinical implications for individualized follow-up and guide therapeutic strategies.

Previous studies that extracted radiomics features from various imaging modalities showed a good AUC in predicting Ki67 status. Fan et al. found that radiomics features extracted from contrast-enhanced CT showed an AUC of 0.726 in predicting Ki-67 status [20]. Feng S et al. extracted radiomics features from dynamic contrast-enhanced magnetic resonance imaging, and their radiomics models reached an AUC of 0.839 (95% confidence interval [CI], 0.768–0.895) within the training set and 0.795 (95% CI, 0.674–0.887) within the independent validation set in the prediction of Ki-67 status [21]. Liu et al. constructed a radiomics model based on ultrasound images, and their results showed an AUC of 0.821 (95% CI:0.764–0.880) and 0.713 (95% CI:0.612–0.814) in the training and validation cohorts [22]. Önner et al. found that textural features extracted from ^18^F-FDG PET/CT are associated with the heterogeneity of BC [23]. Their data showed that the most significant relevant feature was the sphericity in the shape category. For our study, the radiomics features derived from PET/CT images were without shape-based features and expressed as Radscore. Using the Radscore alone, the AUC for the prediction of Ki67 (+) reached 0.83[0.74 0.92] within the training set and 0.75[0.56 0.95] within the testing set.

Some studies reported that integrating clinical features and radiomics features can reach higher accuracy. Wu J et al. combined clinical features and radiomics features derived from ultrasound images, and the combined model showed a higher AUC than the radiomics in the prediction of Ki67 status [24]. In our study, we also conducted a significantly higher accuracy in the combined model, which means the physician’s interpretation is critical besides the radiomics analysis.

This study has limitations. First, the retrospective nature and its small sample size. Second, BC is a heterogeneous disease comprising different molecular sub-types; whether Ki67 can be combined with radiomics features in predicting auxiliary lymph node metastasis and molecular subtypes was also widely discussed.

## Conclusion

The radiomics-derived evaluation score combined with the clinical features could effectively predict Ki67 expression in BC based on PET/CT images.

## Electronic Supplementary Material

Below is the link to the electronic supplementary material.

Supplementary Material 1

Supplementary Material 2

## Data Availability

We confirm that all the materials and data with regard to the analysis in the manuscript are available for request.
