# Supplementary material for: PET/CT Radiomics Integrated with Clinical Indexes as a Tool to Predict Ki67 in Breast Cancer: a Pilot Study
Source: Nucl Med Mol Imaging. 2024 Nov 29;59(3):164–73. doi: 10.1007/s13139-024-00896-9 (PMC12084439; doi:10.1007/s13139-024-00896-9)
Supplement: Supplementary file 1 — Supplementary Material 1 [file 13139_2024_896_MOESM1_ESM.docx]

**Supplementary table** **The categories and the name of all the 704 radiomics features**

| serial number | original feature and the transformations | categories | features' name |
| --- | --- | --- | --- |
| 1 | original feature | shape | Elongation |
| 2 | original feature | shape | Flatness |
| 3 | original feature | shape | Least Axis Length |
| 4 | original feature | shape | Major Axis Length |
| 5 | original feature | shape | Maximum 2D diameter (Column) |
| 6 | original feature | shape | Maximum 2D diameter (Row) |
| 7 | original feature | shape | Maximum 2D diameter (Slice) |
| 8 | original feature | shape | Maximum 3D diameter |
| 9 | original feature | shape | Mesh Volume |
| 10 | original feature | shape | Minor Axis Length |
| 11 | original feature | shape | Sphericity |
| 12 | original feature | shape | Surface Area |
| 13 | original feature | shape | Surface Area to Volume ratio |
| 14 | original feature | shape | Voxel Volume |
| 15 | original feature | First Order | 10th percentile |
| 16 | original feature | First Order | 90th percentile |
| 17 | original feature | First Order | Energy |
| 18 | original feature | First Order | Entropy |
| 19 | original feature | First Order | Interquartile Range |
| 20 | original feature | First Order | Kurtosis |
| 21 | original feature | First Order | Maximum |
| 22 | original feature | First Order | Mean Absolute Deviation |
| 23 | original feature | First Order | Mean |
| 24 | original feature | First Order | Median |
| 25 | original feature | First Order | Minimum |
| 26 | original feature | First Order | Range |
| 27 | original feature | First Order | Robust Mean Absolute Deviation |
| 28 | original feature | First Order | Root Mean Squared |
| 29 | original feature | First Order | Skewness |
| 30 | original feature | First Order | Total Energy |
| 31 | original feature | First Order | Uniformity |
| 32 | original feature | First Order | Variance |
| 33 | original feature | GLRLM | Gray Level Non-Uniformity |
| 34 | original feature | GLRLM | Gray Level Non-Uniformity Normalized |
| 35 | original feature | GLRLM | Gray Level Variance |
| 36 | original feature | GLRLM | High Gray Level Run Emphasis |
| 37 | original feature | GLRLM | Long Run Emphasis |
| 38 | original feature | GLRLM | Long Run High Gray Level Emphasis |
| 39 | original feature | GLRLM | Long Run Low Gray Level Emphasis |
| 40 | original feature | GLRLM | Low Gray Level Run Emphasis |
| 41 | original feature | GLRLM | Run Entropy |
| 42 | original feature | GLRLM | Run Length Non-Uniformity |
| 43 | original feature | GLRLM | Run Length Non-Uniformity Normalized |
| 44 | original feature | GLRLM | Run Percentage |
| 45 | original feature | GLRLM | Run Variance |
| 46 | original feature | GLRLM | Short Run Emphasis |
| 47 | original feature | GLRLM | Short Run High Gray Level Emphasis |
| 48 | original feature | GLRLM | Short Run Low Gray Level Emphasis |
| 49 | original feature | NGTDM | Busyness |
| 50 | original feature | NGTDM | Coarseness |
| 51 | original feature | NGTDM | Complexity |
| 52 | original feature | NGTDM | Contrast |
| 53 | original feature | NGTDM | Strength |
| 54 | original feature | GLDM | Dependence Entropy |
| 55 | original feature | GLDM | Dependence Non-Uniformity |
| 56 | original feature | GLDM | Dependence Non-Uniformity Normalized |
| 57 | original feature | GLDM | Dependence Variance |
| 58 | original feature | GLDM | Gray Level Non-Uniformity |
| 59 | original feature | GLDM | Gray Level Variance |
| 60 | original feature | GLDM | High Gray Level Emphasis |
| 61 | original feature | GLDM | Large Dependence Emphasis |
| 62 | original feature | GLDM | Large Dependence High Gray Level Emphasis |
| 63 | original feature | GLDM | Large Dependence Low Gray Level Emphasis |
| 64 | original feature | GLDM | Low Gray Level Emphasis |
| 65 | original feature | GLDM | Small Dependence Emphasis |
| 66 | original feature | GLDM | Small Dependence High Gray Level Emphasis |
| 67 | original feature | GLDM | Small Dependence Low Gray Level Emphasis |
| 68 | original feature | GLSZM | Gray Level Non-Uniformity |
| 69 | original feature | GLSZM | Gray Level Non-Uniformity Normalized |
| 70 | original feature | GLSZM | Gray Level Variance |
| 71 | original feature | GLSZM | High Gray Level Zone Emphasis |
| 72 | original feature | GLSZM | Large Area Emphasis |
| 73 | original feature | GLSZM | Large Area High Gray Level Emphasis |
| 74 | original feature | GLSZM | Large Area Low Gray Level Emphasis |
| 75 | original feature | GLSZM | Low Gray Level Zone Emphasis |
| 76 | original feature | GLSZM | Size Zone Non-Uniformity |
| 77 | original feature | GLSZM | Size Zone Non-Uniformity Normalized |
| 78 | original feature | GLSZM | Small Area Emphasis |
| 79 | original feature | GLSZM | Small Area High Gray Level Emphasis |
| 80 | original feature | GLSZM | Small Area Low Gray Level Emphasis |
| 81 | original feature | GLSZM | Zone Entropy |
| 82 | original feature | GLSZM | Zone Percentage |
| 83 | original feature | GLSZM | Zone Variance |
| 84 | log-sigma-6-0-mm-3D | First Order | 10th percentile |
| 85 | log-sigma-6-0-mm-3D | First Order | 90th percentile |
| 86 | log-sigma-6-0-mm-3D | First Order | Energy |
| 87 | log-sigma-6-0-mm-3D | First Order | Entropy |
| 88 | log-sigma-6-0-mm-3D | First Order | Interquartile Range |
| 89 | log-sigma-6-0-mm-3D | First Order | Kurtosis |
| 90 | log-sigma-6-0-mm-3D | First Order | Maximum |
| 91 | log-sigma-6-0-mm-3D | First Order | Mean Absolute Deviation |
| 92 | log-sigma-6-0-mm-3D | First Order | Mean |
| 93 | log-sigma-6-0-mm-3D | First Order | Median |
| 94 | log-sigma-6-0-mm-3D | First Order | Minimum |
| 95 | log-sigma-6-0-mm-3D | First Order | Range |
| 96 | log-sigma-6-0-mm-3D | First Order | Robust Mean Absolute Deviation |
| 97 | log-sigma-6-0-mm-3D | First Order | Root Mean Squared |
| 98 | log-sigma-6-0-mm-3D | First Order | Skewness |
| 99 | log-sigma-6-0-mm-3D | First Order | Total Energy |
| 100 | log-sigma-6-0-mm-3D | First Order | Uniformity |
| 101 | log-sigma-6-0-mm-3D | First Order | Variance |
| 102 | log-sigma-6-0-mm-3D | GLRLM | Gray Level Non-Uniformity |
| 103 | log-sigma-6-0-mm-3D | GLRLM | Gray Level Non-Uniformity Normalized |
| 104 | log-sigma-6-0-mm-3D | GLRLM | Gray Level Variance |
| 105 | log-sigma-6-0-mm-3D | GLRLM | High Gray Level Run Emphasis |
| 106 | log-sigma-6-0-mm-3D | GLRLM | Long Run Emphasis |
| 107 | log-sigma-6-0-mm-3D | GLRLM | Long Run High Gray Level Emphasis |
| 108 | log-sigma-6-0-mm-3D | GLRLM | Long Run Low Gray Level Emphasis |
| 109 | log-sigma-6-0-mm-3D | GLRLM | Low Gray Level Run Emphasis |
| 110 | log-sigma-6-0-mm-3D | GLRLM | Run Entropy |
| 111 | log-sigma-6-0-mm-3D | GLRLM | Run Length Non-Uniformity |
| 112 | log-sigma-6-0-mm-3D | GLRLM | Run Length Non-Uniformity Normalized |
| 113 | log-sigma-6-0-mm-3D | GLRLM | Run Percentage |
| 114 | log-sigma-6-0-mm-3D | GLRLM | Run Variance |
| 115 | log-sigma-6-0-mm-3D | GLRLM | Short Run Emphasis |
| 116 | log-sigma-6-0-mm-3D | GLRLM | Short Run High Gray Level Emphasis |
| 117 | log-sigma-6-0-mm-3D | GLRLM | Short Run Low Gray Level Emphasis |
| 118 | log-sigma-6-0-mm-3D | NGTDM | Busyness |
| 119 | log-sigma-6-0-mm-3D | NGTDM | Coarseness |
| 120 | log-sigma-6-0-mm-3D | NGTDM | Complexity |
| 121 | log-sigma-6-0-mm-3D | NGTDM | Contrast |
| 122 | log-sigma-6-0-mm-3D | NGTDM | Strength |
| 123 | log-sigma-6-0-mm-3D | GLDM | Dependence Entropy |
| 124 | log-sigma-6-0-mm-3D | GLDM | Dependence Non-Uniformity |
| 125 | log-sigma-6-0-mm-3D | GLDM | Dependence Non-Uniformity Normalized |
| 126 | log-sigma-6-0-mm-3D | GLDM | Dependence Variance |
| 127 | log-sigma-6-0-mm-3D | GLDM | Gray Level Non-Uniformity |
| 128 | log-sigma-6-0-mm-3D | GLDM | Gray Level Variance |
| 129 | log-sigma-6-0-mm-3D | GLDM | High Gray Level Emphasis |
| 130 | log-sigma-6-0-mm-3D | GLDM | Large Dependence Emphasis |
| 131 | log-sigma-6-0-mm-3D | GLDM | Large Dependence High Gray Level Emphasis |
| 132 | log-sigma-6-0-mm-3D | GLDM | Large Dependence Low Gray Level Emphasis |
| 133 | log-sigma-6-0-mm-3D | GLDM | Low Gray Level Emphasis |
| 134 | log-sigma-6-0-mm-3D | GLDM | Small Dependence Emphasis |
| 135 | log-sigma-6-0-mm-3D | GLDM | Small Dependence High Gray Level Emphasis |
| 136 | log-sigma-6-0-mm-3D | GLDM | Small Dependence Low Gray Level Emphasis |
| 137 | log-sigma-6-0-mm-3D | GLSZM | Gray Level Non-Uniformity |
| 138 | log-sigma-6-0-mm-3D | GLSZM | Gray Level Non-Uniformity Normalized |
| 139 | log-sigma-6-0-mm-3D | GLSZM | Gray Level Variance |
| 140 | log-sigma-6-0-mm-3D | GLSZM | High Gray Level Zone Emphasis |
| 141 | log-sigma-6-0-mm-3D | GLSZM | Large Area Emphasis |
| 142 | log-sigma-6-0-mm-3D | GLSZM | Large Area High Gray Level Emphasis |
| 143 | log-sigma-6-0-mm-3D | GLSZM | Large Area Low Gray Level Emphasis |
| 144 | log-sigma-6-0-mm-3D | GLSZM | Low Gray Level Zone Emphasis |
| 145 | log-sigma-6-0-mm-3D | GLSZM | Size Zone Non-Uniformity |
| 146 | log-sigma-6-0-mm-3D | GLSZM | Size Zone Non-Uniformity Normalized |
| 147 | log-sigma-6-0-mm-3D | GLSZM | Small Area Emphasis |
| 148 | log-sigma-6-0-mm-3D | GLSZM | Small Area High Gray Level Emphasis |
| 149 | log-sigma-6-0-mm-3D | GLSZM | Small Area Low Gray Level Emphasis |
| 150 | log-sigma-6-0-mm-3D | GLSZM | Zone Entropy |
| 151 | log-sigma-6-0-mm-3D | GLSZM | Zone Percentage |
| 152 | log-sigma-6-0-mm-3D | GLSZM | Zone Variance |
| 153 | wavelet-LLH | First Order | 10th percentile |
| 154 | wavelet-LLH | First Order | 90th percentile |
| 155 | wavelet-LLH | First Order | Energy |
| 156 | wavelet-LLH | First Order | Entropy |
| 157 | wavelet-LLH | First Order | Interquartile Range |
| 158 | wavelet-LLH | First Order | Kurtosis |
| 159 | wavelet-LLH | First Order | Maximum |
| 160 | wavelet-LLH | First Order | Mean Absolute Deviation |
| 161 | wavelet-LLH | First Order | Mean |
| 162 | wavelet-LLH | First Order | Median |
| 163 | wavelet-LLH | First Order | Minimum |
| 164 | wavelet-LLH | First Order | Range |
| 165 | wavelet-LLH | First Order | Robust Mean Absolute Deviation |
| 166 | wavelet-LLH | First Order | Root Mean Squared |
| 167 | wavelet-LLH | First Order | Skewness |
| 168 | wavelet-LLH | First Order | Total Energy |
| 169 | wavelet-LLH | First Order | Uniformity |
| 170 | wavelet-LLH | First Order | Variance |
| 171 | wavelet-LLH | GLRLM | Gray Level Non-Uniformity |
| 172 | wavelet-LLH | GLRLM | Gray Level Non-Uniformity Normalized |
| 173 | wavelet-LLH | GLRLM | Gray Level Variance |
| 174 | wavelet-LLH | GLRLM | High Gray Level Run Emphasis |
| 175 | wavelet-LLH | GLRLM | Long Run Emphasis |
| 176 | wavelet-LLH | GLRLM | Long Run High Gray Level Emphasis |
| 177 | wavelet-LLH | GLRLM | Long Run Low Gray Level Emphasis |
| 178 | wavelet-LLH | GLRLM | Low Gray Level Run Emphasis |
| 179 | wavelet-LLH | GLRLM | Run Entropy |
| 180 | wavelet-LLH | GLRLM | Run Length Non-Uniformity |
| 181 | wavelet-LLH | GLRLM | Run Length Non-Uniformity Normalized |
| 182 | wavelet-LLH | GLRLM | Run Percentage |
| 183 | wavelet-LLH | GLRLM | Run Variance |
| 184 | wavelet-LLH | GLRLM | Short Run Emphasis |
| 185 | wavelet-LLH | GLRLM | Short Run High Gray Level Emphasis |
| 186 | wavelet-LLH | GLRLM | Short Run Low Gray Level Emphasis |
| 187 | wavelet-LLH | NGTDM | Busyness |
| 188 | wavelet-LLH | NGTDM | Coarseness |
| 189 | wavelet-LLH | NGTDM | Complexity |
| 190 | wavelet-LLH | NGTDM | Contrast |
| 191 | wavelet-LLH | NGTDM | Strength |
| 192 | wavelet-LLH | GLDM | Dependence Entropy |
| 193 | wavelet-LLH | GLDM | Dependence Non-Uniformity |
| 194 | wavelet-LLH | GLDM | Dependence Non-Uniformity Normalized |
| 195 | wavelet-LLH | GLDM | Dependence Variance |
| 196 | wavelet-LLH | GLDM | Gray Level Non-Uniformity |
| 197 | wavelet-LLH | GLDM | Gray Level Variance |
| 198 | wavelet-LLH | GLDM | High Gray Level Emphasis |
| 199 | wavelet-LLH | GLDM | Large Dependence Emphasis |
| 200 | wavelet-LLH | GLDM | Large Dependence High Gray Level Emphasis |
| 201 | wavelet-LLH | GLDM | Large Dependence Low Gray Level Emphasis |
| 202 | wavelet-LLH | GLDM | Low Gray Level Emphasis |
| 203 | wavelet-LLH | GLDM | Small Dependence Emphasis |
| 204 | wavelet-LLH | GLDM | Small Dependence High Gray Level Emphasis |
| 205 | wavelet-LLH | GLDM | Small Dependence Low Gray Level Emphasis |
| 206 | wavelet-LLH | GLSZM | Gray Level Non-Uniformity |
| 207 | wavelet-LLH | GLSZM | Gray Level Non-Uniformity Normalized |
| 208 | wavelet-LLH | GLSZM | Gray Level Variance |
| 209 | wavelet-LLH | GLSZM | High Gray Level Zone Emphasis |
| 210 | wavelet-LLH | GLSZM | Large Area Emphasis |
| 211 | wavelet-LLH | GLSZM | Large Area High Gray Level Emphasis |
| 212 | wavelet-LLH | GLSZM | Large Area Low Gray Level Emphasis |
| 213 | wavelet-LLH | GLSZM | Low Gray Level Zone Emphasis |
| 214 | wavelet-LLH | GLSZM | Size Zone Non-Uniformity |
| 215 | wavelet-LLH | GLSZM | Size Zone Non-Uniformity Normalized |
| 216 | wavelet-LLH | GLSZM | Small Area Emphasis |
| 217 | wavelet-LLH | GLSZM | Small Area High Gray Level Emphasis |
| 218 | wavelet-LLH | GLSZM | Small Area Low Gray Level Emphasis |
| 219 | wavelet-LLH | GLSZM | Zone Entropy |
| 220 | wavelet-LLH | GLSZM | Zone Percentage |
| 221 | wavelet-LLH | GLSZM | Zone Variance |
| 222 | wavelet-LHL | First Order | 10th percentile |
| 223 | wavelet-LHL | First Order | 90th percentile |
| 224 | wavelet-LHL | First Order | Energy |
| 225 | wavelet-LHL | First Order | Entropy |
| 226 | wavelet-LHL | First Order | Interquartile Range |
| 227 | wavelet-LHL | First Order | Kurtosis |
| 228 | wavelet-LHL | First Order | Maximum |
| 229 | wavelet-LHL | First Order | Mean Absolute Deviation |
| 230 | wavelet-LHL | First Order | Mean |
| 231 | wavelet-LHL | First Order | Median |
| 232 | wavelet-LHL | First Order | Minimum |
| 233 | wavelet-LHL | First Order | Range |
| 234 | wavelet-LHL | First Order | Robust Mean Absolute Deviation |
| 235 | wavelet-LHL | First Order | Root Mean Squared |
| 236 | wavelet-LHL | First Order | Skewness |
| 237 | wavelet-LHL | First Order | Total Energy |
| 238 | wavelet-LHL | First Order | Uniformity |
| 239 | wavelet-LHL | First Order | Variance |
| 240 | wavelet-LHL | GLRLM | Gray Level Non-Uniformity |
| 241 | wavelet-LHL | GLRLM | Gray Level Non-Uniformity Normalized |
| 242 | wavelet-LHL | GLRLM | Gray Level Variance |
| 243 | wavelet-LHL | GLRLM | High Gray Level Run Emphasis |
| 244 | wavelet-LHL | GLRLM | Long Run Emphasis |
| 245 | wavelet-LHL | GLRLM | Long Run High Gray Level Emphasis |
| 246 | wavelet-LHL | GLRLM | Long Run Low Gray Level Emphasis |
| 247 | wavelet-LHL | GLRLM | Low Gray Level Run Emphasis |
| 248 | wavelet-LHL | GLRLM | Run Entropy |
| 249 | wavelet-LHL | GLRLM | Run Length Non-Uniformity |
| 250 | wavelet-LHL | GLRLM | Run Length Non-Uniformity Normalized |
| 251 | wavelet-LHL | GLRLM | Run Percentage |
| 252 | wavelet-LHL | GLRLM | Run Variance |
| 253 | wavelet-LHL | GLRLM | Short Run Emphasis |
| 254 | wavelet-LHL | GLRLM | Short Run High Gray Level Emphasis |
| 255 | wavelet-LHL | GLRLM | Short Run Low Gray Level Emphasis |
| 256 | wavelet-LHL | NGTDM | Busyness |
| 257 | wavelet-LHL | NGTDM | Coarseness |
| 258 | wavelet-LHL | NGTDM | Complexity |
| 259 | wavelet-LHL | NGTDM | Contrast |
| 260 | wavelet-LHL | NGTDM | Strength |
| 261 | wavelet-LHL | GLDM | Dependence Entropy |
| 262 | wavelet-LHL | GLDM | Dependence Non-Uniformity |
| 263 | wavelet-LHL | GLDM | Dependence Non-Uniformity Normalized |
| 264 | wavelet-LHL | GLDM | Dependence Variance |
| 265 | wavelet-LHL | GLDM | Gray Level Non-Uniformity |
| 266 | wavelet-LHL | GLDM | Gray Level Variance |
| 267 | wavelet-LHL | GLDM | High Gray Level Emphasis |
| 268 | wavelet-LHL | GLDM | Large Dependence Emphasis |
| 269 | wavelet-LHL | GLDM | Large Dependence High Gray Level Emphasis |
| 270 | wavelet-LHL | GLDM | Large Dependence Low Gray Level Emphasis |
| 271 | wavelet-LHL | GLDM | Low Gray Level Emphasis |
| 272 | wavelet-LHL | GLDM | Small Dependence Emphasis |
| 273 | wavelet-LHL | GLDM | Small Dependence High Gray Level Emphasis |
| 274 | wavelet-LHL | GLDM | Small Dependence Low Gray Level Emphasis |
| 275 | wavelet-LHL | GLSZM | Gray Level Non-Uniformity |
| 276 | wavelet-LHL | GLSZM | Gray Level Non-Uniformity Normalized |
| 277 | wavelet-LHL | GLSZM | Gray Level Variance |
| 278 | wavelet-LHL | GLSZM | High Gray Level Zone Emphasis |
| 279 | wavelet-LHL | GLSZM | Large Area Emphasis |
| 280 | wavelet-LHL | GLSZM | Large Area High Gray Level Emphasis |
| 281 | wavelet-LHL | GLSZM | Large Area Low Gray Level Emphasis |
| 282 | wavelet-LHL | GLSZM | Low Gray Level Zone Emphasis |
| 283 | wavelet-LHL | GLSZM | Size Zone Non-Uniformity |
| 284 | wavelet-LHL | GLSZM | Size Zone Non-Uniformity Normalized |
| 285 | wavelet-LHL | GLSZM | Small Area Emphasis |
| 286 | wavelet-LHL | GLSZM | Small Area High Gray Level Emphasis |
| 287 | wavelet-LHL | GLSZM | Small Area Low Gray Level Emphasis |
| 288 | wavelet-LHL | GLSZM | Zone Entropy |
| 289 | wavelet-LHL | GLSZM | Zone Percentage |
| 290 | wavelet-LHL | GLSZM | Zone Variance |
| 291 | wavelet-LHH | First Order | 10th percentile |
| 292 | wavelet-LHH | First Order | 90th percentile |
| 293 | wavelet-LHH | First Order | Energy |
| 294 | wavelet-LHH | First Order | Entropy |
| 295 | wavelet-LHH | First Order | Interquartile Range |
| 296 | wavelet-LHH | First Order | Kurtosis |
| 297 | wavelet-LHH | First Order | Maximum |
| 298 | wavelet-LHH | First Order | Mean Absolute Deviation |
| 299 | wavelet-LHH | First Order | Mean |
| 300 | wavelet-LHH | First Order | Median |
| 301 | wavelet-LHH | First Order | Minimum |
| 302 | wavelet-LHH | First Order | Range |
| 303 | wavelet-LHH | First Order | Robust Mean Absolute Deviation |
| 304 | wavelet-LHH | First Order | Root Mean Squared |
| 305 | wavelet-LHH | First Order | Skewness |
| 306 | wavelet-LHH | First Order | Total Energy |
| 307 | wavelet-LHH | First Order | Uniformity |
| 308 | wavelet-LHH | First Order | Variance |
| 309 | wavelet-LHH | GLRLM | Gray Level Non-Uniformity |
| 310 | wavelet-LHH | GLRLM | Gray Level Non-Uniformity Normalized |
| 311 | wavelet-LHH | GLRLM | Gray Level Variance |
| 312 | wavelet-LHH | GLRLM | High Gray Level Run Emphasis |
| 313 | wavelet-LHH | GLRLM | Long Run Emphasis |
| 314 | wavelet-LHH | GLRLM | Long Run High Gray Level Emphasis |
| 315 | wavelet-LHH | GLRLM | Long Run Low Gray Level Emphasis |
| 316 | wavelet-LHH | GLRLM | Low Gray Level Run Emphasis |
| 317 | wavelet-LHH | GLRLM | Run Entropy |
| 318 | wavelet-LHH | GLRLM | Run Length Non-Uniformity |
| 319 | wavelet-LHH | GLRLM | Run Length Non-Uniformity Normalized |
| 320 | wavelet-LHH | GLRLM | Run Percentage |
| 321 | wavelet-LHH | GLRLM | Run Variance |
| 322 | wavelet-LHH | GLRLM | Short Run Emphasis |
| 323 | wavelet-LHH | GLRLM | Short Run High Gray Level Emphasis |
| 324 | wavelet-LHH | GLRLM | Short Run Low Gray Level Emphasis |
| 325 | wavelet-LHH | NGTDM | Busyness |
| 326 | wavelet-LHH | NGTDM | Coarseness |
| 327 | wavelet-LHH | NGTDM | Complexity |
| 328 | wavelet-LHH | NGTDM | Contrast |
| 329 | wavelet-LHH | NGTDM | Strength |
| 330 | wavelet-LHH | GLDM | Dependence Entropy |
| 331 | wavelet-LHH | GLDM | Dependence Non-Uniformity |
| 332 | wavelet-LHH | GLDM | Dependence Non-Uniformity Normalized |
| 333 | wavelet-LHH | GLDM | Dependence Variance |
| 334 | wavelet-LHH | GLDM | Gray Level Non-Uniformity |
| 335 | wavelet-LHH | GLDM | Gray Level Variance |
| 336 | wavelet-LHH | GLDM | High Gray Level Emphasis |
| 337 | wavelet-LHH | GLDM | Large Dependence Emphasis |
| 338 | wavelet-LHH | GLDM | Large Dependence High Gray Level Emphasis |
| 339 | wavelet-LHH | GLDM | Large Dependence Low Gray Level Emphasis |
| 340 | wavelet-LHH | GLDM | Low Gray Level Emphasis |
| 341 | wavelet-LHH | GLDM | Small Dependence Emphasis |
| 342 | wavelet-LHH | GLDM | Small Dependence High Gray Level Emphasis |
| 343 | wavelet-LHH | GLDM | Small Dependence Low Gray Level Emphasis |
| 344 | wavelet-LHH | GLSZM | Gray Level Non-Uniformity |
| 345 | wavelet-LHH | GLSZM | Gray Level Non-Uniformity Normalized |
| 346 | wavelet-LHH | GLSZM | Gray Level Variance |
| 347 | wavelet-LHH | GLSZM | High Gray Level Zone Emphasis |
| 348 | wavelet-LHH | GLSZM | Large Area Emphasis |
| 349 | wavelet-LHH | GLSZM | Large Area High Gray Level Emphasis |
| 350 | wavelet-LHH | GLSZM | Large Area Low Gray Level Emphasis |
| 351 | wavelet-LHH | GLSZM | Low Gray Level Zone Emphasis |
| 352 | wavelet-LHH | GLSZM | Size Zone Non-Uniformity |
| 353 | wavelet-LHH | GLSZM | Size Zone Non-Uniformity Normalized |
| 354 | wavelet-LHH | GLSZM | Small Area Emphasis |
| 355 | wavelet-LHH | GLSZM | Small Area High Gray Level Emphasis |
| 356 | wavelet-LHH | GLSZM | Small Area Low Gray Level Emphasis |
| 357 | wavelet-LHH | GLSZM | Zone Entropy |
| 358 | wavelet-LHH | GLSZM | Zone Percentage |
| 359 | wavelet-LHH | GLSZM | Zone Variance |
| 360 | wavelet-HLL | First Order | 10th percentile |
| 361 | wavelet-HLL | First Order | 90th percentile |
| 362 | wavelet-HLL | First Order | Energy |
| 363 | wavelet-HLL | First Order | Entropy |
| 364 | wavelet-HLL | First Order | Interquartile Range |
| 365 | wavelet-HLL | First Order | Kurtosis |
| 366 | wavelet-HLL | First Order | Maximum |
| 367 | wavelet-HLL | First Order | Mean Absolute Deviation |
| 368 | wavelet-HLL | First Order | Mean |
| 369 | wavelet-HLL | First Order | Median |
| 370 | wavelet-HLL | First Order | Minimum |
| 371 | wavelet-HLL | First Order | Range |
| 372 | wavelet-HLL | First Order | Robust Mean Absolute Deviation |
| 373 | wavelet-HLL | First Order | Root Mean Squared |
| 374 | wavelet-HLL | First Order | Skewness |
| 375 | wavelet-HLL | First Order | Total Energy |
| 376 | wavelet-HLL | First Order | Uniformity |
| 377 | wavelet-HLL | First Order | Variance |
| 378 | wavelet-HLL | GLRLM | Gray Level Non-Uniformity |
| 379 | wavelet-HLL | GLRLM | Gray Level Non-Uniformity Normalized |
| 380 | wavelet-HLL | GLRLM | Gray Level Variance |
| 381 | wavelet-HLL | GLRLM | High Gray Level Run Emphasis |
| 382 | wavelet-HLL | GLRLM | Long Run Emphasis |
| 383 | wavelet-HLL | GLRLM | Long Run High Gray Level Emphasis |
| 384 | wavelet-HLL | GLRLM | Long Run Low Gray Level Emphasis |
| 385 | wavelet-HLL | GLRLM | Low Gray Level Run Emphasis |
| 386 | wavelet-HLL | GLRLM | Run Entropy |
| 387 | wavelet-HLL | GLRLM | Run Length Non-Uniformity |
| 388 | wavelet-HLL | GLRLM | Run Length Non-Uniformity Normalized |
| 389 | wavelet-HLL | GLRLM | Run Percentage |
| 390 | wavelet-HLL | GLRLM | Run Variance |
| 391 | wavelet-HLL | GLRLM | Short Run Emphasis |
| 392 | wavelet-HLL | GLRLM | Short Run High Gray Level Emphasis |
| 393 | wavelet-HLL | GLRLM | Short Run Low Gray Level Emphasis |
| 394 | wavelet-HLL | NGTDM | Busyness |
| 395 | wavelet-HLL | NGTDM | Coarseness |
| 396 | wavelet-HLL | NGTDM | Complexity |
| 397 | wavelet-HLL | NGTDM | Contrast |
| 398 | wavelet-HLL | NGTDM | Strength |
| 399 | wavelet-HLL | GLDM | Dependence Entropy |
| 400 | wavelet-HLL | GLDM | Dependence Non-Uniformity |
| 401 | wavelet-HLL | GLDM | Dependence Non-Uniformity Normalized |
| 402 | wavelet-HLL | GLDM | Dependence Variance |
| 403 | wavelet-HLL | GLDM | Gray Level Non-Uniformity |
| 404 | wavelet-HLL | GLDM | Gray Level Variance |
| 405 | wavelet-HLL | GLDM | High Gray Level Emphasis |
| 406 | wavelet-HLL | GLDM | Large Dependence Emphasis |
| 407 | wavelet-HLL | GLDM | Large Dependence High Gray Level Emphasis |
| 408 | wavelet-HLL | GLDM | Large Dependence Low Gray Level Emphasis |
| 409 | wavelet-HLL | GLDM | Low Gray Level Emphasis |
| 410 | wavelet-HLL | GLDM | Small Dependence Emphasis |
| 411 | wavelet-HLL | GLDM | Small Dependence High Gray Level Emphasis |
| 412 | wavelet-HLL | GLDM | Small Dependence Low Gray Level Emphasis |
| 413 | wavelet-HLL | GLSZM | Gray Level Non-Uniformity |
| 414 | wavelet-HLL | GLSZM | Gray Level Non-Uniformity Normalized |
| 415 | wavelet-HLL | GLSZM | Gray Level Variance |
| 416 | wavelet-HLL | GLSZM | High Gray Level Zone Emphasis |
| 417 | wavelet-HLL | GLSZM | Large Area Emphasis |
| 418 | wavelet-HLL | GLSZM | Large Area High Gray Level Emphasis |
| 419 | wavelet-HLL | GLSZM | Large Area Low Gray Level Emphasis |
| 420 | wavelet-HLL | GLSZM | Low Gray Level Zone Emphasis |
| 421 | wavelet-HLL | GLSZM | Size Zone Non-Uniformity |
| 422 | wavelet-HLL | GLSZM | Size Zone Non-Uniformity Normalized |
| 423 | wavelet-HLL | GLSZM | Small Area Emphasis |
| 424 | wavelet-HLL | GLSZM | Small Area High Gray Level Emphasis |
| 425 | wavelet-HLL | GLSZM | Small Area Low Gray Level Emphasis |
| 426 | wavelet-HLL | GLSZM | Zone Entropy |
| 427 | wavelet-HLL | GLSZM | Zone Percentage |
| 428 | wavelet-HLL | GLSZM | Zone Variance |
| 429 | wavelet-HLH | First Order | 10th percentile |
| 430 | wavelet-HLH | First Order | 90th percentile |
| 431 | wavelet-HLH | First Order | Energy |
| 432 | wavelet-HLH | First Order | Entropy |
| 433 | wavelet-HLH | First Order | Interquartile Range |
| 434 | wavelet-HLH | First Order | Kurtosis |
| 435 | wavelet-HLH | First Order | Maximum |
| 436 | wavelet-HLH | First Order | Mean Absolute Deviation |
| 437 | wavelet-HLH | First Order | Mean |
| 438 | wavelet-HLH | First Order | Median |
| 439 | wavelet-HLH | First Order | Minimum |
| 440 | wavelet-HLH | First Order | Range |
| 441 | wavelet-HLH | First Order | Robust Mean Absolute Deviation |
| 442 | wavelet-HLH | First Order | Root Mean Squared |
| 443 | wavelet-HLH | First Order | Skewness |
| 444 | wavelet-HLH | First Order | Total Energy |
| 445 | wavelet-HLH | First Order | Uniformity |
| 446 | wavelet-HLH | First Order | Variance |
| 447 | wavelet-HLH | GLRLM | Gray Level Non-Uniformity |
| 448 | wavelet-HLH | GLRLM | Gray Level Non-Uniformity Normalized |
| 449 | wavelet-HLH | GLRLM | Gray Level Variance |
| 450 | wavelet-HLH | GLRLM | High Gray Level Run Emphasis |
| 451 | wavelet-HLH | GLRLM | Long Run Emphasis |
| 452 | wavelet-HLH | GLRLM | Long Run High Gray Level Emphasis |
| 453 | wavelet-HLH | GLRLM | Long Run Low Gray Level Emphasis |
| 454 | wavelet-HLH | GLRLM | Low Gray Level Run Emphasis |
| 455 | wavelet-HLH | GLRLM | Run Entropy |
| 456 | wavelet-HLH | GLRLM | Run Length Non-Uniformity |
| 457 | wavelet-HLH | GLRLM | Run Length Non-Uniformity Normalized |
| 458 | wavelet-HLH | GLRLM | Run Percentage |
| 459 | wavelet-HLH | GLRLM | Run Variance |
| 460 | wavelet-HLH | GLRLM | Short Run Emphasis |
| 461 | wavelet-HLH | GLRLM | Short Run High Gray Level Emphasis |
| 462 | wavelet-HLH | GLRLM | Short Run Low Gray Level Emphasis |
| 463 | wavelet-HLH | NGTDM | Busyness |
| 464 | wavelet-HLH | NGTDM | Coarseness |
| 465 | wavelet-HLH | NGTDM | Complexity |
| 466 | wavelet-HLH | NGTDM | Contrast |
| 467 | wavelet-HLH | NGTDM | Strength |
| 468 | wavelet-HLH | GLDM | Dependence Entropy |
| 469 | wavelet-HLH | GLDM | Dependence Non-Uniformity |
| 470 | wavelet-HLH | GLDM | Dependence Non-Uniformity Normalized |
| 471 | wavelet-HLH | GLDM | Dependence Variance |
| 472 | wavelet-HLH | GLDM | Gray Level Non-Uniformity |
| 473 | wavelet-HLH | GLDM | Gray Level Variance |
| 474 | wavelet-HLH | GLDM | High Gray Level Emphasis |
| 475 | wavelet-HLH | GLDM | Large Dependence Emphasis |
| 476 | wavelet-HLH | GLDM | Large Dependence High Gray Level Emphasis |
| 477 | wavelet-HLH | GLDM | Large Dependence Low Gray Level Emphasis |
| 478 | wavelet-HLH | GLDM | Low Gray Level Emphasis |
| 479 | wavelet-HLH | GLDM | Small Dependence Emphasis |
| 480 | wavelet-HLH | GLDM | Small Dependence High Gray Level Emphasis |
| 481 | wavelet-HLH | GLDM | Small Dependence Low Gray Level Emphasis |
| 482 | wavelet-HLH | GLSZM | Gray Level Non-Uniformity |
| 483 | wavelet-HLH | GLSZM | Gray Level Non-Uniformity Normalized |
| 484 | wavelet-HLH | GLSZM | Gray Level Variance |
| 485 | wavelet-HLH | GLSZM | High Gray Level Zone Emphasis |
| 486 | wavelet-HLH | GLSZM | Large Area Emphasis |
| 487 | wavelet-HLH | GLSZM | Large Area High Gray Level Emphasis |
| 488 | wavelet-HLH | GLSZM | Large Area Low Gray Level Emphasis |
| 489 | wavelet-HLH | GLSZM | Low Gray Level Zone Emphasis |
| 490 | wavelet-HLH | GLSZM | Size Zone Non-Uniformity |
| 491 | wavelet-HLH | GLSZM | Size Zone Non-Uniformity Normalized |
| 492 | wavelet-HLH | GLSZM | Small Area Emphasis |
| 493 | wavelet-HLH | GLSZM | Small Area High Gray Level Emphasis |
| 494 | wavelet-HLH | GLSZM | Small Area Low Gray Level Emphasis |
| 495 | wavelet-HLH | GLSZM | Zone Entropy |
| 496 | wavelet-HLH | GLSZM | Zone Percentage |
| 497 | wavelet-HLH | GLSZM | Zone Variance |
| 498 | wavelet-HHL | First Order | 10th percentile |
| 499 | wavelet-HHL | First Order | 90th percentile |
| 500 | wavelet-HHL | First Order | Energy |
| 501 | wavelet-HHL | First Order | Entropy |
| 502 | wavelet-HHL | First Order | Interquartile Range |
| 503 | wavelet-HHL | First Order | Kurtosis |
| 504 | wavelet-HHL | First Order | Maximum |
| 505 | wavelet-HHL | First Order | Mean Absolute Deviation |
| 506 | wavelet-HHL | First Order | Mean |
| 507 | wavelet-HHL | First Order | Median |
| 508 | wavelet-HHL | First Order | Minimum |
| 509 | wavelet-HHL | First Order | Range |
| 510 | wavelet-HHL | First Order | Robust Mean Absolute Deviation |
| 511 | wavelet-HHL | First Order | Root Mean Squared |
| 512 | wavelet-HHL | First Order | Skewness |
| 513 | wavelet-HHL | First Order | Total Energy |
| 514 | wavelet-HHL | First Order | Uniformity |
| 515 | wavelet-HHL | First Order | Variance |
| 516 | wavelet-HHL | GLRLM | Gray Level Non-Uniformity |
| 517 | wavelet-HHL | GLRLM | Gray Level Non-Uniformity Normalized |
| 518 | wavelet-HHL | GLRLM | Gray Level Variance |
| 519 | wavelet-HHL | GLRLM | High Gray Level Run Emphasis |
| 520 | wavelet-HHL | GLRLM | Long Run Emphasis |
| 521 | wavelet-HHL | GLRLM | Long Run High Gray Level Emphasis |
| 522 | wavelet-HHL | GLRLM | Long Run Low Gray Level Emphasis |
| 523 | wavelet-HHL | GLRLM | Low Gray Level Run Emphasis |
| 524 | wavelet-HHL | GLRLM | Run Entropy |
| 525 | wavelet-HHL | GLRLM | Run Length Non-Uniformity |
| 526 | wavelet-HHL | GLRLM | Run Length Non-Uniformity Normalized |
| 527 | wavelet-HHL | GLRLM | Run Percentage |
| 528 | wavelet-HHL | GLRLM | Run Variance |
| 529 | wavelet-HHL | GLRLM | Short Run Emphasis |
| 530 | wavelet-HHL | GLRLM | Short Run High Gray Level Emphasis |
| 531 | wavelet-HHL | GLRLM | Short Run Low Gray Level Emphasis |
| 532 | wavelet-HHL | NGTDM | Busyness |
| 533 | wavelet-HHL | NGTDM | Coarseness |
| 534 | wavelet-HHL | NGTDM | Complexity |
| 535 | wavelet-HHL | NGTDM | Contrast |
| 536 | wavelet-HHL | NGTDM | Strength |
| 537 | wavelet-HHL | GLDM | Dependence Entropy |
| 538 | wavelet-HHL | GLDM | Dependence Non-Uniformity |
| 539 | wavelet-HHL | GLDM | Dependence Non-Uniformity Normalized |
| 540 | wavelet-HHL | GLDM | Dependence Variance |
| 541 | wavelet-HHL | GLDM | Gray Level Non-Uniformity |
| 542 | wavelet-HHL | GLDM | Gray Level Variance |
| 543 | wavelet-HHL | GLDM | High Gray Level Emphasis |
| 544 | wavelet-HHL | GLDM | Large Dependence Emphasis |
| 545 | wavelet-HHL | GLDM | Large Dependence High Gray Level Emphasis |
| 546 | wavelet-HHL | GLDM | Large Dependence Low Gray Level Emphasis |
| 547 | wavelet-HHL | GLDM | Low Gray Level Emphasis |
| 548 | wavelet-HHL | GLDM | Small Dependence Emphasis |
| 549 | wavelet-HHL | GLDM | Small Dependence High Gray Level Emphasis |
| 550 | wavelet-HHL | GLDM | Small Dependence Low Gray Level Emphasis |
| 551 | wavelet-HHL | GLSZM | Gray Level Non-Uniformity |
| 552 | wavelet-HHL | GLSZM | Gray Level Non-Uniformity Normalized |
| 553 | wavelet-HHL | GLSZM | Gray Level Variance |
| 554 | wavelet-HHL | GLSZM | High Gray Level Zone Emphasis |
| 555 | wavelet-HHL | GLSZM | Large Area Emphasis |
| 556 | wavelet-HHL | GLSZM | Large Area High Gray Level Emphasis |
| 557 | wavelet-HHL | GLSZM | Large Area Low Gray Level Emphasis |
| 558 | wavelet-HHL | GLSZM | Low Gray Level Zone Emphasis |
| 559 | wavelet-HHL | GLSZM | Size Zone Non-Uniformity |
| 560 | wavelet-HHL | GLSZM | Size Zone Non-Uniformity Normalized |
| 561 | wavelet-HHL | GLSZM | Small Area Emphasis |
| 562 | wavelet-HHL | GLSZM | Small Area High Gray Level Emphasis |
| 563 | wavelet-HHL | GLSZM | Small Area Low Gray Level Emphasis |
| 564 | wavelet-HHL | GLSZM | Zone Entropy |
| 565 | wavelet-HHL | GLSZM | Zone Percentage |
| 566 | wavelet-HHL | GLSZM | Zone Variance |
| 567 | wavelet-HHH | First Order | 10th percentile |
| 568 | wavelet-HHH | First Order | 90th percentile |
| 569 | wavelet-HHH | First Order | Energy |
| 570 | wavelet-HHH | First Order | Entropy |
| 571 | wavelet-HHH | First Order | Interquartile Range |
| 572 | wavelet-HHH | First Order | Kurtosis |
| 573 | wavelet-HHH | First Order | Maximum |
| 574 | wavelet-HHH | First Order | Mean Absolute Deviation |
| 575 | wavelet-HHH | First Order | Mean |
| 576 | wavelet-HHH | First Order | Median |
| 577 | wavelet-HHH | First Order | Minimum |
| 578 | wavelet-HHH | First Order | Range |
| 579 | wavelet-HHH | First Order | Robust Mean Absolute Deviation |
| 580 | wavelet-HHH | First Order | Root Mean Squared |
| 581 | wavelet-HHH | First Order | Skewness |
| 582 | wavelet-HHH | First Order | Total Energy |
| 583 | wavelet-HHH | First Order | Uniformity |
| 584 | wavelet-HHH | First Order | Variance |
| 585 | wavelet-HHH | GLRLM | Gray Level Non-Uniformity |
| 586 | wavelet-HHH | GLRLM | Gray Level Non-Uniformity Normalized |
| 587 | wavelet-HHH | GLRLM | Gray Level Variance |
| 588 | wavelet-HHH | GLRLM | High Gray Level Run Emphasis |
| 589 | wavelet-HHH | GLRLM | Long Run Emphasis |
| 590 | wavelet-HHH | GLRLM | Long Run High Gray Level Emphasis |
| 591 | wavelet-HHH | GLRLM | Long Run Low Gray Level Emphasis |
| 592 | wavelet-HHH | GLRLM | Low Gray Level Run Emphasis |
| 593 | wavelet-HHH | GLRLM | Run Entropy |
| 594 | wavelet-HHH | GLRLM | Run Length Non-Uniformity |
| 595 | wavelet-HHH | GLRLM | Run Length Non-Uniformity Normalized |
| 596 | wavelet-HHH | GLRLM | Run Percentage |
| 597 | wavelet-HHH | GLRLM | Run Variance |
| 598 | wavelet-HHH | GLRLM | Short Run Emphasis |
| 599 | wavelet-HHH | GLRLM | Short Run High Gray Level Emphasis |
| 600 | wavelet-HHH | GLRLM | Short Run Low Gray Level Emphasis |
| 601 | wavelet-HHH | NGTDM | Busyness |
| 602 | wavelet-HHH | NGTDM | Coarseness |
| 603 | wavelet-HHH | NGTDM | Complexity |
| 604 | wavelet-HHH | NGTDM | Contrast |
| 605 | wavelet-HHH | NGTDM | Strength |
| 606 | wavelet-HHH | GLDM | Dependence Entropy |
| 607 | wavelet-HHH | GLDM | Dependence Non-Uniformity |
| 608 | wavelet-HHH | GLDM | Dependence Non-Uniformity Normalized |
| 609 | wavelet-HHH | GLDM | Dependence Variance |
| 610 | wavelet-HHH | GLDM | Gray Level Non-Uniformity |
| 611 | wavelet-HHH | GLDM | Gray Level Variance |
| 612 | wavelet-HHH | GLDM | High Gray Level Emphasis |
| 613 | wavelet-HHH | GLDM | Large Dependence Emphasis |
| 614 | wavelet-HHH | GLDM | Large Dependence High Gray Level Emphasis |
| 615 | wavelet-HHH | GLDM | Large Dependence Low Gray Level Emphasis |
| 616 | wavelet-HHH | GLDM | Low Gray Level Emphasis |
| 617 | wavelet-HHH | GLDM | Small Dependence Emphasis |
| 618 | wavelet-HHH | GLDM | Small Dependence High Gray Level Emphasis |
| 619 | wavelet-HHH | GLDM | Small Dependence Low Gray Level Emphasis |
| 620 | wavelet-HHH | GLSZM | Gray Level Non-Uniformity |
| 621 | wavelet-HHH | GLSZM | Gray Level Non-Uniformity Normalized |
| 622 | wavelet-HHH | GLSZM | Gray Level Variance |
| 623 | wavelet-HHH | GLSZM | High Gray Level Zone Emphasis |
| 624 | wavelet-HHH | GLSZM | Large Area Emphasis |
| 625 | wavelet-HHH | GLSZM | Large Area High Gray Level Emphasis |
| 626 | wavelet-HHH | GLSZM | Large Area Low Gray Level Emphasis |
| 627 | wavelet-HHH | GLSZM | Low Gray Level Zone Emphasis |
| 628 | wavelet-HHH | GLSZM | Size Zone Non-Uniformity |
| 629 | wavelet-HHH | GLSZM | Size Zone Non-Uniformity Normalized |
| 630 | wavelet-HHH | GLSZM | Small Area Emphasis |
| 631 | wavelet-HHH | GLSZM | Small Area High Gray Level Emphasis |
| 632 | wavelet-HHH | GLSZM | Small Area Low Gray Level Emphasis |
| 633 | wavelet-HHH | GLSZM | Zone Entropy |
| 634 | wavelet-HHH | GLSZM | Zone Percentage |
| 635 | wavelet-HHH | GLSZM | Zone Variance |
| 636 | wavelet-LLL | First Order | 10th percentile |
| 637 | wavelet-LLL | First Order | 90th percentile |
| 638 | wavelet-LLL | First Order | Energy |
| 639 | wavelet-LLL | First Order | Entropy |
| 640 | wavelet-LLL | First Order | Interquartile Range |
| 641 | wavelet-LLL | First Order | Kurtosis |
| 642 | wavelet-LLL | First Order | Maximum |
| 643 | wavelet-LLL | First Order | Mean Absolute Deviation |
| 644 | wavelet-LLL | First Order | Mean |
| 645 | wavelet-LLL | First Order | Median |
| 646 | wavelet-LLL | First Order | Minimum |
| 647 | wavelet-LLL | First Order | Range |
| 648 | wavelet-LLL | First Order | Robust Mean Absolute Deviation |
| 649 | wavelet-LLL | First Order | Root Mean Squared |
| 650 | wavelet-LLL | First Order | Skewness |
| 651 | wavelet-LLL | First Order | Total Energy |
| 652 | wavelet-LLL | First Order | Uniformity |
| 653 | wavelet-LLL | First Order | Variance |
| 654 | wavelet-LLL | GLRLM | Gray Level Non-Uniformity |
| 655 | wavelet-LLL | GLRLM | Gray Level Non-Uniformity Normalized |
| 656 | wavelet-LLL | GLRLM | Gray Level Variance |
| 657 | wavelet-LLL | GLRLM | High Gray Level Run Emphasis |
| 658 | wavelet-LLL | GLRLM | Long Run Emphasis |
| 659 | wavelet-LLL | GLRLM | Long Run High Gray Level Emphasis |
| 660 | wavelet-LLL | GLRLM | Long Run Low Gray Level Emphasis |
| 661 | wavelet-LLL | GLRLM | Low Gray Level Run Emphasis |
| 662 | wavelet-LLL | GLRLM | Run Entropy |
| 663 | wavelet-LLL | GLRLM | Run Length Non-Uniformity |
| 664 | wavelet-LLL | GLRLM | Run Length Non-Uniformity Normalized |
| 665 | wavelet-LLL | GLRLM | Run Percentage |
| 666 | wavelet-LLL | GLRLM | Run Variance |
| 667 | wavelet-LLL | GLRLM | Short Run Emphasis |
| 668 | wavelet-LLL | GLRLM | Short Run High Gray Level Emphasis |
| 669 | wavelet-LLL | GLRLM | Short Run Low Gray Level Emphasis |
| 670 | wavelet-LLL | NGTDM | Busyness |
| 671 | wavelet-LLL | NGTDM | Coarseness |
| 672 | wavelet-LLL | NGTDM | Complexity |
| 673 | wavelet-LLL | NGTDM | Contrast |
| 674 | wavelet-LLL | NGTDM | Strength |
| 675 | wavelet-LLL | GLDM | Dependence Entropy |
| 676 | wavelet-LLL | GLDM | Dependence Non-Uniformity |
| 677 | wavelet-LLL | GLDM | Dependence Non-Uniformity Normalized |
| 678 | wavelet-LLL | GLDM | Dependence Variance |
| 679 | wavelet-LLL | GLDM | Gray Level Non-Uniformity |
| 680 | wavelet-LLL | GLDM | Gray Level Variance |
| 681 | wavelet-LLL | GLDM | High Gray Level Emphasis |
| 682 | wavelet-LLL | GLDM | Large Dependence Emphasis |
| 683 | wavelet-LLL | GLDM | Large Dependence High Gray Level Emphasis |
| 684 | wavelet-LLL | GLDM | Large Dependence Low Gray Level Emphasis |
| 685 | wavelet-LLL | GLDM | Low Gray Level Emphasis |
| 686 | wavelet-LLL | GLDM | Small Dependence Emphasis |
| 687 | wavelet-LLL | GLDM | Small Dependence High Gray Level Emphasis |
| 688 | wavelet-LLL | GLDM | Small Dependence Low Gray Level Emphasis |
| 689 | wavelet-LLL | GLSZM | Gray Level Non-Uniformity |
| 690 | wavelet-LLL | GLSZM | Gray Level Non-Uniformity Normalized |
| 691 | wavelet-LLL | GLSZM | Gray Level Variance |
| 692 | wavelet-LLL | GLSZM | High Gray Level Zone Emphasis |
| 693 | wavelet-LLL | GLSZM | Large Area Emphasis |
| 694 | wavelet-LLL | GLSZM | Large Area High Gray Level Emphasis |
| 695 | wavelet-LLL | GLSZM | Large Area Low Gray Level Emphasis |
| 696 | wavelet-LLL | GLSZM | Low Gray Level Zone Emphasis |
| 697 | wavelet-LLL | GLSZM | Size Zone Non-Uniformity |
| 698 | wavelet-LLL | GLSZM | Size Zone Non-Uniformity Normalized |
| 699 | wavelet-LLL | GLSZM | Small Area Emphasis |
| 700 | wavelet-LLL | GLSZM | Small Area High Gray Level Emphasis |
| 701 | wavelet-LLL | GLSZM | Small Area Low Gray Level Emphasis |
| 702 | wavelet-LLL | GLSZM | Zone Entropy |
| 703 | wavelet-LLL | GLSZM | Zone Percentage |
| 704 | wavelet-LLL | GLSZM | Zone Variance |

Note: GLRLM, gray-level run length matrix; NGTDM, neighboring gray-tone difference matrix; GLDM, gray-level dependence matrix; GLSZM, gray-level size zone matrix.
