# Supplementary material for: PET/CT Radiomics Integrated with Clinical Indexes as a Tool to Predict Ki67 in Breast Cancer: a Pilot Study
Source: Nucl Med Mol Imaging. 2024 Nov 29;59(3):164–73. doi: 10.1007/s13139-024-00896-9 (PMC12084439; doi:10.1007/s13139-024-00896-9)
Supplement: Supplementary file 2 — Supplementary Material 2 [file 13139_2024_896_MOESM2_ESM.docx]

**Supplementary table 2 Comparisons of eight radiomics features between two groups**

| eight radiomics features | Ki67- (n = 35) | Ki67+ (n = 79) | Z/*t* | *P* |
| --- | --- | --- | --- | --- |
| log-sigma-6-0-mm-3D_GLRLM_ShortRunHighGrayLevelEmphasis | 433.801 (384.600, 465.811) | 387.683 (321.771, 429.328) | -3.081a | **0.002** |
| wavelet-LHL_GLRLM_RunEntropy | 4.507 ± 0.247 | 4.410 ± 0.307 | 1.658 | 0.100 |
| wavelet-LHH_GLDM_DependenceVariance | 0.974 (0.645, 1.317) | 1.450 (0.796, 3.063) | -2.516a | **0.012** |
| wavelet-LHH_GLSZM_SmallAreaLowGrayLevelEmphasis | 0.024 (0.017, 0.038) | 0.032 (0.019, 0.043) | 1.035a | 0.301 |
| wavelet-HLL_GLSZM_SmallAreaEmphasis | 0.758 ± 0.080 | 0.732 ± 0.077 | 1.608 | 0.111 |
| wavelet-HHL_NGTDM_Contrast | 0.709 (0.382, 1.661) | 0.500 (0.265, 1.177) | 1.705a | 0.088 |
| wavelet-HHH_GLSZM_GrayLevelNonUniformityNormalized | 0.064 ± 0.012 | 0.060 ± 0.008 | 2.071 | **0.041** |
| wavelet-LLL_first order_90Percentile | 13.618 (12.050, 14.782) | 14.707 (13.358, 14.865) | 1.834a | 0.067 |

Note: GLRLM, gray-level run length matrix; NGTDM, neighboring gray-tone difference matrix; GLDM, gray-level dependence matrix; GLSZM, gray-level size zone matrix; a, Z test.
